# Evidence against PALB2 involvement in Icelandic breast cancer susceptibility

**DOI:** 10.1186/1477-5751-7-5

**Published:** 2008-07-17

**Authors:** Haukur Gunnarsson, Adalgeir Arason, Elizabeth M Gillanders, Bjarni A Agnarsson, Gudrun Johannesdottir, Oskar Th Johannsson, Rosa B Barkardottir

**Affiliations:** 1 Department of Pathology, Landspitali – University Hospital, Reykjavik, Iceland; 2 Inherited Disease Research Branch, National Human Genome Research Institute, NIH, Baltimore, Maryland, USA; 3 Department of Oncology, Landspitali – University Hospital, Reykjavik, Iceland

## Abstract

Several mutations in the *PALB2 *gene (partner and localizer of *BRCA2*) have been associated with an increased risk of breast cancer, including a founder mutation, 1592delT, reported in Finnish breast cancer families. Although most often the risk is moderate, it doesn't exclude families with high-risk mutations to exist and such observations have been reported. To see if high-risk *PALB2*-mutations may be present in the geographically confined population of Iceland, linkage analysis was done on 111 individuals, thereof 61 breast cancer cases, from 9 high-risk non-*BRCA1*/*BRCA2 *breast cancer families, targeting the *PALB2 *region. Also, screening for the 1592delT founder mutation in the 9 high-risk families and in 638 unselected breast cancer cases was performed. The results indicate no linkage in any of the high-risk families and screening for the 1592delT mutation was negative in all samples. *PALB2 *appears not to be a significant factor in high-risk breast cancer families in Iceland and the 1592delT mutation is not seen to be associated with breast cancer in Iceland.

## Background

Breast cancer is among the most frequent human cancers and the most common carcinoma in women in the Western world, where one out of every ten women is affected. A dominant pattern of inheritance is evident in approximately 5–10% of all breast cancers. To date, two main breast cancer susceptibility genes have been identified; *BRCA1 *and *BRCA2 *accounting for nearly half of high-incidence breast cancer families and an increased relative risk of breast cancer by 10- to 20- fold [1,2]. Other known breast cancer susceptibility genes such as *CHEK2 *and *ATM *have a more moderate penetrance with an increased lifetime risk of about 2- to 3- fold [2].

The *PALB2 *gene is a *BRCA2 *binding factor that ensures *BRCA2 *function as a tumor suppressor and has been shown to cause Fanconi anemia subtype FA-N when biallelic germ-line mutations occur in the gene [3-5]. Recent studies have reported several mutations in *PALB2 *to be associated with an increased risk of breast cancer [6-9]. One is the founder mutation 1592delT which has been found to be present at a significantly elevated frequency in breast cancer families in Finland, resulting in a 4-fold increased risk to mutation carriers [6]. Although predisposing *PALB2 *mutations generally appear to cause moderate risk of breast cancer [8], mutations have also been found in strong hereditary breast cancer families [7,9] and might thus be worthwhile searching for by linkage analysis in e.g. geographically confined populations.

Only two *BRCA1 *and *BRCA2 *mutations have been found in the Icelandic population, *BRCA2 *999del5 and *BRCA1 *G5193A, both being founder mutations explaining a large proportion of familial breast cancer in Iceland [10]. The *BRCA2 *999del5 mutation is much more frequent, accounting for around 40% of the hereditary cases and found in about 8% of unselected breast cancer cases and 0,4% of population based control [11]. A *BRCA2 *999del5 mutation is also the most frequently occurring *BRCA1/2 *mutation in Finland [12], and haplotype analysis of Finnish and Icelandic *BRCA2 *999del5 families did not exclude the possibility of a common ancient origin of the mutation [13]. The *BRCA1 *G5193A mutation however is very rare, found in less than 2,5% of hereditary breast cancer families and in 0,2% of unselected breast cancer cases [14]. The extreme frequency figures of these two founder mutations reflect low genetic diversity in the Icelandic population. The Icelandic population is a very young one, the country being settled by a few thousand founders about 1100 years ago. Low genetic diversity is probably explained by the relatively homogeneous group of settlers, and genetic drift resulting from repeated population bottlenecks due to diseases and famines [15,16].

The aim of this study is to find out if the PALB2 gene is likely to play a significant role in breast cancer susceptibility in Iceland, by linkage analysis of high-risk non-BRCA1/2 breast cancer families with markers closely surrounding the PALB2 gene to test the possibility of a highly penetrant mutation, and by screening for the Finnish 1592delT founder mutation in the family members and a large group of unselected breast cancer patients.

## Results and Discussion

No evidence of linkage in the *PALB2 *region was found in any of the high risk breast cancer families [see Additional file 1]. The two markers flanking the *PALB2 *gene, D16S420 (~0.58 Mb centromeric to *PALB2*) and D16S412 (~0.45 Mb telomeric to *PALB2*), showed highly negative Lod scores of total -4.18 (θ = 0,00001) and -2.69 (θ = 0,00001).

Several *PALB2 *mutations have been identified and one of them has been found as a recurrent mutation, 1592delT in Finland [6]. In light of the possible common ancient origin of the *BRCA2 *999del5 mutation in the Finnish and the Icelandic population we decided to screen for the Finnish *PALB2 *founder mutation, 1592delT, in Icelandic breast cancer cases. The mutation was not detected in 638 unselected breast cancer cases nor in any of the 111 family members of the nine high risk breast cancer families.

The results of this study suggest that *PALB2 *is not a significant contributor to breast cancer in high-risk breast cancer families in Iceland. Furthermore, the results show that the 1592delT mutation appears not to be associated with breast cancer in the Icelandic population, and if occurring it would be very rare. However the results can not exclude the possibility that other *PALB2 *mutations causing low or moderate breast cancer risk exist in the Icelandic population. To determine that, it would be necessary to perform a *PALB2 *mutation screening or SNP analysis in a large cohort of breast cancer cases.

## Methods

### Study population

The sample set consisted of: 111 individuals from nine high-risk non-*BRCA1*/*BRCA2 *breast cancer families (Table 1), 38 controls to evaluate allele frequencies in the linkage analysis, and 638 unselected breast cancer cases diagnosed in the period 1987–2003. All patients contributing to the research have given their informed consent. The research has been approved by the Icelandic Data Projection Authority and the National Bioethics Committee.

**Table 1:** Summary of the main clinical characteristics of the 9 high risk breast cancer families

**Family**	**Number of ** **affected** ** individuals**	**Number of typed** **individuals** ** (affected)**	**Mean age at first** **diagnosis** ** years (range)**
1	5	6(4)	61.8(44-76)
2	5	11(5)	49.4 (44–64)
3*	10	14 (10)	57.4 (50–88)
4*	9	17 (8)	54.2 (38–75)
5*	11	20 (9)	49.7 (29–70)
6	6	11 (5)	57.5 (35–79)
7	8	8 (6)	61.1 (42–79)
8	5	11 (5)	49.6 (30–66)
9	9	13 (9)	51.8 (30–77)

**Total**	**68**	**111 (61)**	**54.6 (29–88)**

*Co-occurrence of ovarian cancer in the family (one family member diagnosed).

### Laboratory Analysis

Blood samples were lyzed and DNA extracted from nuclei according to Miller et al [17]. DNA from paraffin-embedded tissue was extracted as described by Jönsson et al [18] and from fresh-frozen tissue using the Wizard Genomic DNA Purification Kit (Promega).

To evaluate linkage in the families the following microsatellite markers were used: D16S3036, D16S412, D16S420 and D16S3068 (primer sequences are available in the UCSC Genome Browser, genome assembly March 2006) [19]. Primers to screen for the 1592delT mutation were as in Erkko et al [6]. All the primers were purchased from MWG Biotech and each forward primer was 5' labeled with either FAM or HEX fluorophore reporter. The PCR conditions for all primer pairs were: 3 minutes incubation at 94°C, followed by 35 cycles of 94°C for 30 seconds, 55°C for 45 seconds, 72°C for 45 seconds and a final extension at 72°C for 10 minutes, except for the 1592delT primers for which the annealing temperature was 64°C instead of 55°C. The PCR products were analysed using an automated ABI PRISM 3130 × l Genetic Analyzer, alleles were called automatically using the GeneMapper software v4.0 and checked manually.

### Statistical analysis

The Genetic Analysis System software (GAS) was used to check the genotyping data for Mendelian inconsistencies within the families. Genotypes which were inconsistent with Mendelian inheritance were reviewed. Any ambiguous genotypes were dropped. Allele frequencies for the microsatellite markers were calculated using founder individuals from the families as well as the control individuals using the Gconvert program [20]. Evidence for linkage was evaluated using parametric linkage analysis methods. Two-point LOD scores were calculated using the FASTLINK program, assuming a rare (frequency = 0.0033) dominantly inherited disease allele. Age-dependant liability classes were defined using the modified CASH model [21,22].

## Abbreviations

*PALB2: *Partner and localizer of *BRCA*; *BRCA1: *Breast cancer susceptibility gene 1; *BRCA2: *Breast cancer susceptibility gene 2; *CHEK2: CHK2 *checkpoint homolog (S. pombe);  *ATM: *Ataxia telangiectasia mutated.

## Competing interests

The authors declare that they have no competing interests.

## Authors' contributions

HG conducted the genotyping, wrote the manuscript and participated in the statistical analysis and design of the study. AA did the pedigree analysis, participated in the design of the study and drafting of the manuscript. EMG performed the statistical analysis, participated in the pedigree analysis and in the design of the study. BAA provided biological samples and pathological information. GJ participated in handling of the DNA samples. OThJ participated in the recruiting of the cases. RBB initiated the study and coordinated it, and participated in the design and drafting of the manuscript. All authors read and approved the manuscript.

## Supplementary Material

Additional file 1 Two-point Lod scores for microsatellite markers surrounding the PALB2 gene* at recombination fractions (θ) 0.00001, 0.01 and 0.05 (gender averaged). Click here for file
